# Three Years with the Army of the Potomac—A Personal Military History

**Published:** 1912-04

**Authors:** William Warren Potter

**Affiliations:** Buffalo, N. Y., Brevet Lieutenant Colonel, U. S. Volunteers; Surgeon in Charge of First Division Field Hospital Second Army Corps; Surgeon 57th Regiment New York Volunteers; Assistant Surgeon 49th Regiment New York Volunteers; Recorder Second Division Hospital Sixth Army Corps, etc., etc.


### Three Years with the Army of the Potomac—A Personal Military History

By William Warren Potter, M.D., Buffalo, N. Y., Brevet Lieutenant Colonel, U. S. Volunteers; Surgeon in Charge of First Division Field Hospital Second Army Corps; Surgeon 57th Regiment New York Volunteers; Assistant Surgeon 49th Regiment New York Volunteers; Recorder Second Division Hospital Sixth Army Corps, etc., etc.

### At Mitchell's Station.

Saturday, September 19, 1863, I started for the front this morning, reaching Culpepper about five o'clock P. M., where I remained over night. On the following morning, Sunday, I joined the command near Mitchell's Station, which is the last R. R. station before the O. & A. Railway crosses the Rapidan. Our camp is near the Rapidan River, and the rebel camps are in plain sight of my tent on the heights opposite. We are ten miles from our last camp at Morrisville. I am still with the hospital though we have very few sick, most of them having been sent away before the move.

September 22. We are in plain view of the rebel batteries on the opposite side of the Rapidan, and from their position they could shell our camps if they saw fit. The cavalry has been fighting on our right, apparently near Madison C. H. We have orders for eight days' rations and to be in readiness to move at a moment's notice, which looks like work soon.

September 25. Our sick were sent off today to Culpepper C. H.—another indication of a move. One of the enemy's signal stations is plainly visible, and I see their flags waving at all hours of the day. I have a Richmond paper of the 23d, which says Longstreet's and Hill's Corps are out west opposing Rosecrans, and claiming a great victory for their side at Chickamauga on the 19th. The 11th and 12th Corps from this army have been sent west to reinforce Rosecrans, which reduces our status to a strict defensive.

September 27. We don't move yet, but the indications are that we shall soon fall back towards Alexandria. The enemy is on the alert, feeling our flanks, and may precipitate a move at any time; at all events, there is much mystery in the situation as it appears to me.

September 30. Still waiting and hourly expecting, but no definite orders to move as yet. I have been two years in the service, the 2nd anniversary of my muster-in having occurred the day I left home two weeks ago. One year yet remains before my term expires, and who knows what it will bring forth? Time alone can answer the question, and it only remains for me to do my duty to the best of my ability, trusting the result to Him who "Doeth all things well."

October 2. It has rained steadily for the past twelve hours, and altogether it is one of the most disagreeable days I have known for a long time. Yesterday we had a little excitement in the way of a horse race at Mitchell's Station, between a horse owned by Captain Rose, Commissary of the Third Brigade, and one belonging to the staff of Corps Headquarters. Colonel Smith, Chief Commissary of the Corps, and myself were the judges, and Rose's horse won by half a neck. The rebels looked on from the heights opposite, quiet and interested spectators. Today a man was shot in this Division for desertion—the first execution in the First Division. I have had my horse mustered into service at a value of $225.00, so that I can recover in case he is killed. We have been troubled for supplies for our mess since we have been here, but tonight we are to dine upon roast pork, mashed potatoes, onions, etc.

October 4. Today has been a beautiful day—in marked contrast to the weather lately, and especially to Friday last.

October 7. We were relieved on the 5th from our uncomfortable and dangerous position at the front by the 6th Corps, and yesterday retired, to Culpepper C. H., where we are now encamped. It is raining quite hard again, and we have just received orders to send away our sick tomorrow, which looks like activity again. Dr. Letterman, Medical Director of the Army of the Potomac, goes North tomorrow to be married.

October 10. We moved our camp a short distance yesterday, and are again ordered to move immediately.

### Auburn and Bristoe.

October 11. Sunday, five o'clock P. M. We have been on the march since twelve o'clock last night, and have just reached Bealton Station on the north side of the Rappahannock. The army has evacuated Culpepper by this time, as the last train on the O. & A. R. R. has just passed here for Alexandria. What the movement signifies I cannot say, but it looks as if the enemy was upon our right flank, and we would retreat towards Alexandria in the morning.

October 16. We have been constantly on the move since Monday, and fighting a good part of the time. The Army of the Potomac is back at Centerville, and the 2d Corps was the rear guard during the movement. It appears that Lee's army got upon our right flank, as was intimated probable a little way back, and we were obliged to march fast, fight a good deal, and manœuvre still more to save ourselves and our trains; but we did it and, besides, made the enemy pay dear for the trouble it put us too. On Monday, the 12th, we recrossed the Rappahannock, moving from Bealton to Brandy Station, but found no great force of the enemy there, as was expected. We came back and bivouacked at Bealton late that night, and Tuesday morning moved in the direction of Warrenton. At nine o'clock P. M. we bivouacked in a cornfield near Auburn, not over 400 yards from the enemy's pickets, building large fires to deceive them as to our numbers, and starting next morning at daylight, with the expectation of reaching Catlett's Station in two or three hours. The enemy, however, planted a battery across our path, and shelled the First Division while cooking coffee at Auburn. I had a large load of sick in the ambulances, and they shelled our train pretty severely, driving back the cavalry on our flank. I thought at one time our whole train would be captured, but the First Division charged and drove off the rascals in season to save it. General Warren now came along to the head of the train, and gave orders for it to give way to let a battery pass, which was soon put into action and did good work. I was obliged to unload the sick and take on the wounded, which I brought safely to Centerville after dark. We passed Catlett's Station near noon, where I saw General Meade, staff, and escort drawn up in line.

At Bristoe, just at nightfall, we had another encounter with the enemy, who sought to cut off our retreat by getting possession of our road, where it intersects the one they were moving on. But the 2nd Division was pushed forward, seizing the R. R. cut which served as a breastwork, and gave them a terrible threshing. We captured five guns, two colors, and over four hundred prisoners, retiring afterwards to Centerville under cover of the darkness.

I was busy all night operating and dressing the wounded, with but one Assistant Surgeon, relying upon the hospital stewards and nurses for further aid. I amputated an arm for a cavalry captain about two o'clock A. M., who thought he could not endure the suffering until morning. Next day, Thursday, the 15th, we were ordered to send the wounded to Fairfax Station for shipment to Washington, and one poor fellow, Rose of the 57th, who was suffering so from shock that we thought best not to operate upon him, must now receive attention; so, upon Dr. Dougherty's advice, I amputated his right thigh and left arm, four men holding a rubber blanket over the table to keep the rain off, while the operation was being made. The train had gone on, but I kept one ambulance back to take him, and after the operation he was put carefully aboard, given half a glass of brandy, and sent on to overtake the others. Just as the ambulance was moving off he asked: "Doctor, you think I'll get well now, don't you?" And, sure enough, he did recover. This case is reported in the "Medical and Surgical History of the War," Part Second, Surgical Volume, Page 611, and Part Third, Surgical Volume, Page 253.

Today, Friday, 16th, I am not well from an attack of colic. It has rained ever since yesterday when I was operating on Rose. The Corps is in line of battle, and everything looks gloomy enough.

Sunday, October 18. We have been here at Centerville since Wednesday night, but I fancy we'll not stay much longer. The movements of the past week have given the Second Corps new honors, and General Meade has complimented us in orders. Today has been the warmest and pleasantest day we have had for a long time.

October 21. Near Auburn. I was in the saddle thirteen hours yesterday. We left Centerville Monday morning (the 19th) early, in a terrific rain storm, and marched to Bristoe Station, where one of our battles was fought a week ago today. Yesterday we moved up here, which is about half way between Catlett's Station and Warrenton. The exact spot where we are now encamped is the scene of our first battle of this day week, when the rebels attacked my train early in the morning. Night before last it was reported that Hill's and Ewell's corps were at Warrenton, and last night it was reported that all the rebel forces had recrossed the Rappahannock about two hours before we arrived here. I am inclined to credit this last report, as we are not moving today, as we probably would be were they this side of the river. They have entirely destroyed the R. R. from Bristoe Station to Warrenton Junction, and I presume as far as the Rappahannock too. The day is very warm, but I feel almost sick with a sore throat. I took cold at Centerville, and am still suffering from its effects. I saw Custer on the march yesterday at the head of his Cavalry Brigade, clad in velvet and gold lace, his locks waving in the breeze—a perfect Murat in manner and appearance.

October 24. We moved about five miles on the 22nd, and are now encamped on the Warrenton R. R., midway between Warrenton and Warrenton Junction. I went up to Warrenton day before yesterday on a pleasure ride, where I saw the 6th Corps. They always appear to get a good place somehow, but have less actual fighting than the 2nd Corps. Division hospitals have been temporarily abandoned, but I still retain charge of the ambulances and the ambulance Corps.

I have been appointed one of the chief operating surgeons of the Division (of whom there are three), which, however, will not interfere with my charge of the ambulance corps. I presume this appointment was made on account of my conduct at Centerville. The Medical Director, Dr. A. N. Dougherty, spoke in complimentary terms thereof, not only to me personally, but to others who have mentioned it to me since. My appointment is to fill a vacancy occasioned by the resignation of Surgeon C. S. Wood, 66th New York, who has been appointed Medical Director, Department of the Pacific, with headquarters at San Francisco.

October 26. Yesterday we changed our camp a short distance to get out of the mud, and now have a fine place in a dry pine grove. The weather today is like a northern autumn. We have just received instructions to pack up and get ready to move at a moment's notice.

October 28. We did not move day before yesterday as expected, and are still here in the pine grove. Today we are having a fireplace built in our tent, so we expect to be comfortable tonight with an indoor fire. My cold is not well, but am better.

October 30. We captured a camera at Morrisville last summer, and a few days ago obtained chemicals from Washington, and are now in full blast taking tintypes, which affords entertainment, as well as useful employment. Our fireplace is a success, the chimney does not smoke—a wonder—and everybody is happy. Dr. Brower Gesner, of the Third Division Ambulance Corps, is chief photographer, and I act as assistant. Captain Livermore, chief of ambulances for the Corps, Lieutenant Pelton, of the Third Division train, and two officers of the Second Division Corps, are with us every day, sitting for their pictures in all sorts of groups and positions. A photograph in my military album shows a group of the ambulance officers, and it was copied from one of the tintypes that we took at this time.

Saturday, October 31. The weather is very fine, and we are quite comfortable with our fireplace to warm us at evening. The month closes with the uncertainty of a possible movement of the army at any time, but in comparative quietude for the moment.

